# Follow the calcium road: Conserved mechanisms of growth and development in *Marchantia polymorpha*

**DOI:** 10.1093/plphys/kiae622

**Published:** 2024-11-22

**Authors:** Jiawen Chen, Erin Cullen

**Affiliations:** Plant Physiology, American Society of Plant Biologists; Division of Crop Biotechnics, Department of Biosystems, KU Leuven, Leuven 3001, Belgium; Plant Physiology, American Society of Plant Biologists

Calcium (Ca^2+^) signalling in plants is essential for the perception of mechanical cues such as touch, gravity, turgor pressure, and cell elongation. Plant cells generally actively maintain low concentrations of Ca^2+^ in the cytoplasm ([Ca^2+^]_cyt_), which generates a concentration gradient between organelles and the cytoplasm. This means that when Ca^2+^ enters the cytoplasm, [Ca^2+^]_cyt_ concentration spikes, creating a calcium wave and translating stress into intracellular electric and ionic signals. These signals can then be transduced into physiological responses, including cell division, growth, and development.

Five groups of mechanosensitive channels have been characterized in plants. One such protein is MID1-COMPLEMENTING ACTIVITY (MCA), a land plant-specific mechanosensitive ion channel (Nishii et al. 2021). MCA proteins are plasma membrane localized and have been characterized in multiple plant taxa. MCA1 was first identified in the dicot *Arabidopsis thaliana*, where it mediates Ca^2+^ influx and root touch sensitivity (Nakagawa et al. 2007). More severe phenotypes are reported in monocots. In rice, *mca* mutants exhibit developmental defects and decreased growth in rice cell cultures (Kurusu et al. 2012). In maize, mutants show strong developmental defects, which can be linked to impaired cell division and differentiation (Rosa et al. 2017). Therefore, MCAs may be involved in a Ca^2+^ signaling pathway that regulates growth and development.

In this issue of *Plant Physiology*, Iwano et al. (2024) identified a single MCA ortholog in the model liverwort *Marchantia polymorpha* and examined the relationship between MpMCA and cell division in both vegetative and reproductive development. The authors examined the localization of MpMCA using transcriptional reporters and discovered that expression is spatially correlated with the apical notch of the thallus (vegetative tissue), as well as the female archegonia and male antheridia (reproductive organs). These are regions that are known to possess high levels of cell proliferation.

To examine the link between MpMCA and Ca^2+^ signaling, the authors imaged a FRET-based Ca^2+^ sensor in vivo in transgenic *M. polymorpha* lines (Nagai et al. 2004). Interestingly, a strong calcium signal was observed in the wild-type apical notch, whereas signal was diminished in the Mp*mca* mutant (Fig. 1A). Similarly, the calcium signal was higher in the archegonia and antheridia of wild-type lines compared with the Mp*mca* mutant. Complementation with Mp*MCA* restored the calcium phenotype in all tissues. These data indicate that MpMCA may regulate [Ca^2+^]_cyt_ levels in areas with prolific cell division in both vegetative and reproductive organs.

**Figure 1.**
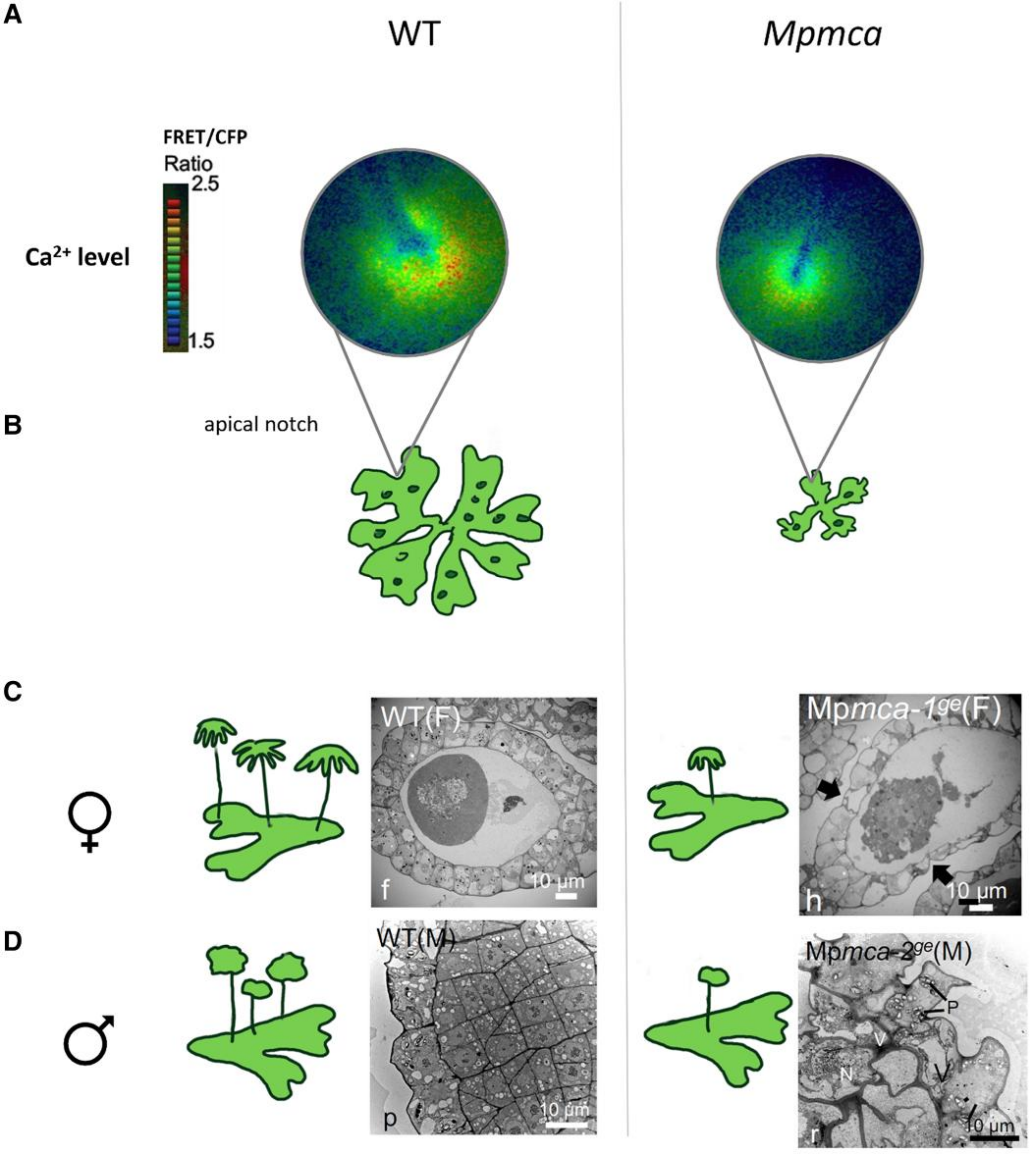
MpMCA controls Ca^2+^ levels during cell proliferation in vegetative thallus and reproductive tissues. **A)** [Ca^2+^]_cyt_ levels in wild type and *Mpmca* mutant visualized with a FRET/CFP sensor, represented with the FRET/CFP ratio, excited at 430 nm. Images from 3-day old gemmalings. **B)** Schematic of the phenotype of wild-type and *Mpmca* mutant thallus tissue: smaller thallus and fewer gemma cups in mutant. **C)** Schematic of the phenotype of archegoniophore formation in wild type and *Mpmca*, with underdeveloped archegoniophores in the mutant, accompanied by transmission electron microscopy (TEM) images of an archegonia section. Arrows indicate cells surrounding the egg cells. **D)** Same as **C)** for antheridiophores (schematic) and antheridia (TEM images). Adapted from Iwano et al. (2024).

To continue investigating MpMCA function, the authors characterized the growth and developmental phenotypes of Mp*mca* mutants generated through CRISPR/Cas9 genome editing. In both male and female mutant lines, thalli exhibited defects such as reduced surface area and fewer gemma cups (Fig. 1B). These defects were rescued by complementation. Two complementary cell proliferation assays (a thymidine analog EdU incorporation assay and observation of MpCYCD1 expression) provide evidence that cell division occurs in the apical notch of wild-type *M. polymorpha* and is significantly reduced in the Mp*mca* mutant. Therefore, the phenotypic defects described in Mp*mca* mutants might be linked to decreased levels of cell proliferation. The authors confirmed that MpMCA was polarly localized to the plasma membrane in apical notch cells, using a translational reporter. Intriguingly, structures resembling lipid bodies were observed through transmission electron microscopy in the apical notch of wild-type thalli, yet far fewer of these structures were observed in mutant thalli. This suggests a potential link between MpMCA and lipid metabolism.

The Mp*mca* mutant also showed impaired development in reproductive structures, exhibiting abnormal morphology of egg cells in the archegonia (Fig. 1C) and sperm cells in the antheridia (Fig. 1D). Advanced imaging of antheridia development in Mp*mca* revealed that cell division is disrupted and suggests that MpMCA may regulate cell division during spermatogenesis. The Mp*mca* mutant also exhibited a delay in the production and number of archegoniophores and antheridiophores. Fertilization of archegoniophores produced by Mp*mca* with wild-type sperm was largely unsuccessful, and sperm release was defective in Mp*mca*. Taken together, these data indicate that MpMCA is required for development of reproductive structures in *M. polymorpha*. Impressively, *A. thaliana MCA1* is largely capable of complementing the Mp*mca* mutant phenotype, indicating that MCA function may be conserved between *A. thaliana* and *M. polymorpha*.

To test the role of MCA in external stress tolerance, calcium signal was imaged in wild type and Mp*mca* during gemmae hydration and germination. A significant increase in the calcium signal was observed after 15 h in wild type. The authors applied mannitol to induce hyperosmotic stress, which changes turgor pressure, and also observed an increase in the calcium signal. Thus, MCA regulates calcium in the presence of osmotic stress.

To conclude, this paper suggests a conserved role for Ca^2+^ signaling via MpMCA in land plants. Orthologous MCA proteins have roles in cell proliferation in vascular plants. This work reveals that MpMCA similarly controls cell proliferation throughout the *M. polymorpha* life cycle under both stressed and unstressed conditions. These results suggest that MpMCA may be acting as a part of a mechanosensitive Ca^2+^ channel complex. Future electrophysiology and pharmacological experiments will provide direct evidence of Ca^2+^ transport. Finally, this paper demonstrates that MpMCA plays a role in the sensing of osmotic stress, and future work could explore the role of MpMCA in perception of temperature, gravitational, and light stress.

## Data Availability

No data were generated or analyzed in this study.
